# Sex differences in central adiposity, hemodynamic, and respiratory parameters among healthy young Nigerian adults: a cross-sectional study

**DOI:** 10.3389/fphys.2026.1949970

**Published:** 2026-10-28

**Authors:** Jovita Eghe Egharevba, Mark Chidi Enyinnaya, Clinton Nwanaga Uche, Gerald Olwit, Grace Acomo, James Nelson Okema

**Affiliations:** 1 Department of Human Physiology, Federal University of Technology Owerri (FUTO), Owerri, Imo State, Nigeria; 2 Department of Human Physiology, College of Health Sciences, University of Port Harcourt, Choba, Rivers State, Nigeria; 3 Department of Internal Medicine, Kabale University School of Medicine, Kabale, Uganda; 4 Department of Internal Medicine, St. Mary’s Hospital Lacor, Gulu, Uganda; 5 Faculty of Medicine, Gulu University, Gulu, Uganda

**Keywords:** blood pressure, breath-holding time, central adiposity, sex differences, sub-Saharan Africa, waist-to-height ratio, waist-to-hip ratio, young adults

## Abstract

**Background:**

Body mass index does not capture regional fat distribution, which is now recognised as a stronger determinant of cardiometabolic and pulmonary function than total adiposity. Whether these associations are already detectable in apparently healthy young adults, and whether they extend to respiratory endurance, remains poorly characterised in sub-Saharan African populations.

**Methods:**

In a cross-sectional study of 81 apparently healthy undergraduates (45 male, 36 females; aged 18–30 years) at the Federal University of Technology, Owerri, Nigeria, we measured waist circumference, waist-to-hip ratio (WHR), waist-to-height ratio (WHtR) and conicity index (ConI) alongside systolic blood pressure (SBP), diastolic blood pressure (DBP), mean arterial pressure (MAP), resting heart rate (HR), and breath-holding time (BHT). Between-sex comparisons used independent-samples t-tests, corroborated by Mann-Whitney U tests after Shapiro-Wilk and Levene’s testing indicated departures from normality for several variables. Associations were examined using Spearman’s rank correlation, with sex-stratified analysis, exact coefficients and 95% confidence intervals (bootstrap, 10,000 resamples), and a significance threshold of p < 0.05. Multiplicity was controlled with the Benjamini-Hochberg false discovery rate (FDR, q < 0.05) applied consistently across both the nine between-sex comparisons and the correlation family; key associations were additionally tested by partial correlation adjusting for age and body mass index (BMI).

**Results:**

Waist circumference did not differ by sex (80.54 ± 8.04 cm vs 79.00 ± 8.41 cm, p = 0.403), but WHR was significantly higher in males (0.85 ± 0.04 vs 0.80 ± 0.05, p < 0.001, surviving correction), indicating a more android fat distribution despite equivalent waist girth. Males had nominally higher SBP (122.04 ± 7.82 vs 118.25 ± 7.13 mmHg, p = 0.027) and longer BHT (43.02 ± 13.15 vs 36.94 ± 13.91 s, p = 0.047), neither surviving FDR correction (q = 0.076 and q = 0.092, respectively), whereas males had significantly lower resting HR than females at both thresholds (70.96 ± 10.68 vs 78.56 ± 11.40 bpm, p = 0.003, q = 0.014). Mann-Whitney U tests confirmed all between-sex conclusions except for WHtR, which reached nominal significance non-parametrically (U test p = 0.038) but not by t-test (p = 0.057); neither survived FDR correction. HR correlated positively with SBP in males (ρ = 0.325, nominal p = 0.030) but not females, and did not survive correction (q = 0.201). WHR correlated modestly and positively with BHT in males (ρ = 0.294, nominal p = 0.050), which likewise did not survive correction (q = 0.201) and weakened further after adjustment for age and BMI (partial ρ = 0.279, p = 0.063); no adiposity index correlated with BHT in females.

**Conclusion:**

Even where absolute waist circumference is equivalent between sexes, WHR was robustly higher in males, consistent with a more android pattern of fat distribution. Sex differences in SBP and BHT, and a correlation between WHR and BHT in males, were observed at nominal significance but did not survive correction for multiple comparisons or adjustment for age and BMI; these findings are hypothesis-generating and require confirmation in an adequately powered study with objective pulmonary function testing before any claim of a sex-specific adiposity–respiratory relationship can be made. WHR may add information beyond BMI and waist circumference alone for early cardiometabolic screening, but this cross-sectional, single-centre dataset cannot establish the direction or mechanism of any association.

## 1 Introduction

Cardiovascular disease remains a leading cause of morbidity and mortality worldwide, and evidence increasingly points to early, subclinical physiological alterations that precede overt disease by decades (Arnett et al., 2019). Central, or visceral, adiposity has emerged as a more discriminating correlate of cardiometabolic risk than total body weight, because visceral fat is metabolically active, secreting pro-inflammatory cytokines and activating the renin–angiotensin–aldosterone system in ways that promote vasoconstriction, endothelial dysfunction, and sympathetic overactivity (Grassi et al., 1995; Engeli et al., 2005; Hall et al., 2015). Body mass index, still the most widely used screening tool, does not distinguish fat distribution from fat mass, and a substantial proportion of individuals with a “normal” body mass index nonetheless carry excess abdominal fat – a pattern sometimes termed normal-weight central obesity (Wildman et al., 2008).

Anthropometric indices such as waist circumference, waist-to-hip ratio (WHR), waist-to-height ratio (WHtR), and the conicity index (ConI) have each been proposed as low-cost alternatives or adjuncts to body mass index for capturing this risk (Janssen et al., 2004; Ashwell et al., 2012; Ashwell and Gibson, 2016). Large prospective cohorts continue to support this: in the UK Biobank, each 5% increment in WHtR was associated with approximately 20% higher risk of ischaemic cardiovascular disease independent of body-fat percentage (Feng et al., 2024), WHR outperforms body mass index for reclassifying cardiovascular risk across multiple outcomes (Butt et al., 2023), and a recent meta-analysis of over 700,000 participants confirmed a dose-dependent association between WHR and myocardial infarction risk (Zhang et al., 2024). Their comparative performance, however, appears to vary by population and by sex, and few studies have examined them jointly against both cardiovascular and respiratory outcomes within the same apparently healthy cohort. Most existing evidence linking adiposity to impaired lung mechanics comes from older or already-obese populations assessed by spirometry, a resource that remains inconsistently available in many low- and middle-income clinical settings; whether a simple, equipment-free respiratory measure such as breath-holding time can detect early, adiposity-related respiratory change in lean, healthy young adults before spirometric access or overt obesity are relevant is therefore of direct practical interest for respiratory screening in resource-limited settings.

Respiratory breath-holding time (BHT) is an underused but simple, non-invasive index of integrated cardiopulmonary and autonomic function, reflecting oxygen reserve, carbon dioxide tolerance, and central and peripheral chemoreceptor sensitivity (American Thoracic Society, 1995; Skow et al., 2015; Trembach and Zabolotskikh, 2018). Central adiposity is hypothesised to reduce breath-holding capacity through mechanical restriction of diaphragmatic excursion and reduced chest-wall compliance (Salome et al., 2010); in men with obesity, breath-holding duration is indeed shorter and is predicted jointly by WHR and peripheral chemosensitivity to carbon dioxide (Trembach and Zabolotskikh, 2018). It remains untested, however, whether this obesity-range, mechanically mediated relationship generalises to lean, healthy young adults, in whom breath-holding duration is understood to reflect a more complex interplay of chemical, mechanical, and volitional drives (Bosco et al., 2004; Skow et al., 2015), and whether any such association is sex-specific.

Young adults in university settings across sub-Saharan Africa already show a measurable and, in some settings, rising burden of central adiposity: a four-year time-series study of Cameroonian university students documented rising WHR in men and rising abdominal obesity in women over the observation period, with waist circumference and body mass index independently predicting prevalent hypertension even at this life stage (Choukem et al., 2017). In a broader South African adult sample, central obesity by waist-to-hip or waist-to-height ratio was present in over half of participants, and roughly a third of those with an ostensibly normal body mass index nonetheless met criteria for normal-weight central obesity (Owolabi et al., 2017), underscoring that anthropometric screening tools such as WHR may carry independent information not captured by body mass index across the region generally, not only in this cohort. Whether comparable, early sex-differentiated patterns extend to respiratory endurance in similarly young, apparently healthy West African cohorts has not, to our knowledge, been examined.

We therefore examined the association of four central adiposity indices with hemodynamic parameters and breath-holding time in a cohort of apparently healthy young Nigerian university students, with the specific aims of (i) comparing adiposity, hemodynamic, and respiratory parameters between sexes; (ii) determining which adiposity index correlates most strongly with cardiovascular and respiratory measures; and (iii) testing whether these associations differ between males and females.

## 2 Methods

### 2.1 Study design and setting

This was a descriptive cross-sectional study conducted among undergraduate students at the Federal University of Technology, Owerri (FUTO), Nigeria.

### 2.2 Participants

Eighty-one apparently healthy students aged 18–30 years (45 male, 36 female) were recruited using random/systematic sampling from the eligible undergraduate population. Eligible participants were apparently healthy, willing to provide informed consent, and able to complete all study measurements. Individuals with known cardiovascular or respiratory disease, hypertension or antihypertensive medication use, asthma or other lung conditions, pregnancy, or current smoking or regular alcohol use were excluded. The sample size of 81 was determined pragmatically by the number of eligible, consenting students accessible within the study period rather than by an *a priori* power calculation; this is acknowledged as a limitation (Section 4.1). All 81 students who were approached and met the eligibility criteria agreed to participate and completed the full study protocol; there was no refusal, withdrawal, or non-participation at any stage of recruitment or data collection.

### 2.3 Anthropometric measurements

Waist circumference was measured with a flexible tape at the midpoint between the lowest rib and the iliac crest, at the end of a normal expiration, to the nearest 0.1 cm. Hip circumference was measured at the widest point of the buttocks. Height was measured barefoot using a stadiometer and weight using a calibrated scale in light clothing. WHR was calculated as waist circumference divided by hip circumference, and WHtR as waist circumference divided by height. Body mass index (BMI) was calculated as weight (kg) divided by the square of height (m²). The conicity index (ConI) was calculated as ConI = WC/[0.109 × √(weight/height)], where waist circumference (WC) is expressed in metres and weight and height in kilograms and metres respectively, following the method of Valdez (1991). Waist and hip circumference measurements followed World Health Organization protocol recommendations (World Health Organization, 2011).

The conicity index is denoted *ConI* throughout this manuscript to avoid confusion with the abbreviation for confidence interval, which is used extensively in **Tables 1**–**3**.

**Table 1:** Between-sex comparison of anthropometric, hemodynamic, and respiratory parameters.

Variable	Male (n = 45)	Female (n = 36)	Nominal p-value (FDR q-value)	Cohen’s d [95% CI of mean difference, native units]
Waist circumference, cm	80.54 ± 8.04	79.00 ± 8.41	0.403 (0.403)	0.19 (−2.11, 5.19)
Waist-to-hip ratio	0.85 ± 0.04	0.80 ± 0.05	<0.001 (<0.001)	0.86 (0.02, 0.06)
Waist-to-height ratio	0.454 ± 0.044	0.475 ± 0.051	0.057 (0.093)	−0.43 (−0.04, 0.00)
Conicity index (ConI)	1.19 ± 0.07	1.20 ± 0.09	0.352 (0.396)	−0.21 (−0.05, 0.02)
Systolic blood pressure, mmHg	122.04 ± 7.82	118.25 ± 7.13	0.027 (0.076)	0.50 (0.44, 7.14)
Diastolic blood pressure, mmHg	81.04 ± 8.75	78.11 ± 7.12	0.108 (0.139)	0.36 (−0.66, 6.52)
Mean arterial pressure, mmHg	94.71 ± 7.97	91.49 ± 6.61	0.055 (0.091)	0.44 (−0.07, 6.51)
Resting heart rate, bpm	70.96 ± 10.68	78.56 ± 11.40	0.003 (0.014)	−0.69 (−12.50, −2.70)
Breath-holding time, s	43.02 ± 13.15	36.94 ± 13.91	0.047 (0.092)	0.45 (0.08, 12.08)

Benjamini-Hochberg FDR-adjusted significance (family of nine comparisons, q < 0.05): WHR and resting heart rate remain significant (q < 0.05); systolic blood pressure, breath-holding time, waist-to-height ratio, and mean arterial pressure are nominally significant or borderline but do not survive correction (q = 0.08–0.09); waist circumference, conicity index, and diastolic blood pressure are not significant at either threshold. The 95% confidence interval reported alongside each Cohen’s d value bounds the raw between-sex mean difference in that variable’s native units, not the standardised effect size d itself. Cohen’s d and the mean difference are both expressed as male minus female throughout, such that a positive value indicates a higher mean in males and a negative value indicates a higher mean in females.

**Table 2:** Normality, variance homogeneity, and non-parametric sensitivity analysis (new).

Variable	Shapiro-Wilk p (Male)	Shapiro-Wilk p (Female)	Levene’s p	Mann-Whitney U test p	FDR q (Mann-Whitney)
Waist circumference, cm	0.010	0.320	0.694	0.527	0.527
Waist-to-hip ratio	0.828	0.243	0.365	0.001	0.008
Waist-to-height ratio	0.108	0.861	0.300	0.038	0.085
Conicity index (ConI)	0.898	0.049	0.743	0.170	0.218
Systolic blood pressure, mmHg	0.002	0.097	0.871	0.025	0.075
Diastolic blood pressure, mmHg	0.013	0.019	0.406	0.221	0.248
Mean arterial pressure, mmHg	0.066	0.702	0.501	0.105	0.157
Resting heart rate, bpm	0.043	0.475	0.516	0.003	0.014
Breath-holding time, s	0.002	0.189	0.718	0.051	0.092

Shapiro-Wilk and Mann-Whitney U tests performed separately within each sex/between sexes on the raw participant-level dataset. Bold interpretation: p < 0.05 for Shapiro-Wilk indicates a significant departure from normality for that sex-variable combination. Levene’s test was centred on the median. FDR q-values for the Mann-Whitney U tests were computed by the Benjamini-Hochberg procedure across the same nine-comparison family as **Table 1**, for comparability.

**Table 3:** Spearman correlations, sex-stratified, with exact coefficients, p-values, bootstrap 95% CIs, and FDR correction (replaces the previous, incompletely-reported **Table 2**).

a. Internal-consistency checks – definitional correlations, not part of the corrected hypothesis-testing family						
Correlation	Sex		n	ρ (rho)	p	95% CI (bootstrap)
WHtR – waist circumference	Male		45	0.902	<0.001	(0.797, 0.954)
WHtR – waist circumference	Female		36	0.924	<0.001	(0.841, 0.961)
WHtR – BMI	Male		45	0.710	<0.001	(0.492, 0.852)
WHtR – BMI	Female		36	0.735	<0.001	(0.506, 0.858)
WHtR – hip circumference	Male		45	0.745	<0.001	(0.527, 0.886)
WHtR – hip circumference	Female		36	0.616	<0.001	(0.342, 0.804)
b. Hypothesis-testing family (14 tests; Benjamini-Hochberg FDR applied, q < 0.05)						
Correlation	Sex	n	ρ (rho)	Nominal p	95% CI (bootstrap)	FDR q
WHR – WHtR	Male	45	0.285	0.057	(−0.014, 0.546)	0.201
WHR – WHtR	Female	36	0.670	<0.001	(0.427, 0.829)	<0.001
Heart rate – systolic BP	Male	45	0.325	0.030	(0.032, 0.570)	0.201
Heart rate – systolic BP	Female	36	0.111	0.519	(−0.284, 0.458)	0.661
Heart rate – diastolic BP	Male	45	0.194	0.201	(−0.109, 0.470)	0.463
Heart rate – diastolic BP	Female	36	0.171	0.319	(−0.202, 0.514)	0.463
WHR – breath-holding time	Male	45	0.294	0.050	(−0.025, 0.588)	0.201
WHR – breath-holding time	Female	36	−0.167	0.331	(−0.471, 0.169)	0.463
WC – breath-holding time	Male	45	−0.056	0.716	(−0.364, 0.243)	0.734
WC – breath-holding time	Female	36	−0.059	0.734	(−0.373, 0.282)	0.734
WHtR – breath-holding time	Male	45	−0.073	0.635	(−0.389, 0.238)	0.734
WHtR – breath-holding time	Female	36	−0.199	0.245	(−0.498, 0.146)	0.463
ConI – breath-holding time	Male	45	0.182	0.233	(−0.144, 0.475)	0.463
ConI – breath-holding time	Female	36	−0.178	0.299	(−0.484, 0.168)	0.463

Spearman’s ρ with exact two-tailed nominal p-values; 95% confidence intervals obtained by bias-corrected bootstrap resampling (10,000 resamples) of paired observations. FDR q-values computed by the Benjamini-Hochberg procedure across the fourteen-test family in panel (b); the six definitional correlations in panel (a) are not included in this correction, per the rationale given in Section 2.6. WC, waist circumference; WHR, waist-to-hip ratio; WHtR, waist-to-height ratio; ConI, conicity index; BP, blood pressure.

### 2.4 Hemodynamic measurements

Blood pressure and heart rate were measured after five minutes of seated rest using a manual sphygmomanometer and stethoscope, with the arm supported at heart level. Two readings were taken two minutes apart and averaged. Heart rate was recorded by 60-second radial pulse palpation. Pulse pressure was calculated as systolic minus diastolic pressure and mean arterial pressure as diastolic pressure plus one-third of pulse pressure.

### 2.5 Respiratory breath-holding time

Following a normal inspiration, participants held their breath for as long as comfortably tolerated. All participants received identical, standardized verbal instructions read from a fixed script prior to each trial. Participants were explicitly instructed to stop the breath-hold immediately if they experienced dizziness, chest discomfort, or any other distress, and the examiner monitored each participant throughout the trial and was authorised to halt it at any time on safety grounds. Two trials separated by a two-minute rest were performed, and the longer duration was recorded to the nearest second using a stopwatch.

We note explicitly that breath-holding after a normal (tidal) inspiration, as used here, is methodologically distinct from protocols that standardise breath-holding at total lung capacity (maximal inspiration) or at functional residual capacity (FRC), as recommended for some standardised respiratory testing (American Thoracic Society, 1995). Because lung volume at the point of breath-holding onset is itself a determinant of breath-holding duration (Bosco et al., 2004; Skow et al., 2015), this unstandardised starting lung volume introduces intra- and inter-individual variance that is independent of chemoreceptor or adiposity-related effects; we return to this point in the Limitations (Section 4.1).

### 2.6 Statistical analysis

Data were analysed in SPSS (version 25) and cross-verified in Python (SciPy 1.x, statsmodels), with the raw dataset re-examined in full for this revision. There were no missing data for any of the anthropometric, hemodynamic, or respiratory variables among the 81 participants analysed, so no method for handling missing data was required.

Continuous variables are presented as mean ± standard deviation. Prior to between-sex comparison, normality of each of the nine continuous variables (**Table 1**) was assessed within each sex using the Shapiro-Wilk test, and homogeneity of variance was assessed using Levene’s test centred on the median. Independent-samples t-tests (equal variances assumed, as Levene’s test was non-significant for all nine variables; **Table 2**) were used as the primary between-sex comparison. Because Shapiro-Wilk testing indicated significant departures from normality for several variables in one or both sexes (waist circumference and breath-holding time in males; conicity index, systolic and diastolic blood pressure in one or both sexes; **Table 2**), the Mann-Whitney U test was additionally performed for all nine variables as a distribution-free sensitivity analysis, per the reviewer’s request.

Associations between adiposity indices and hemodynamic or respiratory variables were assessed using Spearman’s rank correlation coefficient, performed separately for male and female participants, with exact coefficients (ρ), exact nominal p-values, and 95% confidence intervals obtained by bias-corrected bootstrap resampling (10,000 resamples). We distinguish two families of correlation tests. The first comprises the correlations of WHtR with waist circumference, BMI, and hip circumference: because WHtR is calculated directly from waist circumference, and shares a further determinant (height/weight) with BMI, these correlations are expected to be strong by mathematical construction rather than by independent empirical association, and are reported only as internal-consistency checks on the dataset, not as hypothesis tests requiring multiplicity correction. The second, genuine hypothesis-testing family comprises the remaining fourteen correlations reported in **Table 3** (WHR with WHtR; heart rate with systolic and diastolic blood pressure; and each of the four adiposity indices with breath-holding time; each tested separately by sex).

For both families of comparisons – the nine between-sex comparisons (**Table 1**) and the fourteen-test correlation family (**Table 3**) – we applied the Benjamini-Hochberg false discovery rate (FDR) procedure at q = 0.05 (Benjamini and Hochberg, 1995), in preference to a Bonferroni correction, because several of the variables involved (WHR, WHtR, and ConI in the between-sex family; the adiposity-BHT correlations, which share the same four indices, in the correlation family) are not statistically independent of one another, and FDR control is robust under this kind of positive dependence while remaining less conservative than Bonferroni for a correlated test family. This represents a change from the previous draft, in which a Bonferroni correction was applied to the correlation family; that approach had been adopted because exact p-values were not available for the full correlation set at the time. With the complete raw dataset now re-analysed, exact statistics are available for every comparison, and we therefore apply FDR consistently across both families for methodological coherence.

For the associations of greatest interest – the WHR–BHT correlation in males and the heart rate–systolic blood pressure correlation in males – we additionally computed partial Spearman correlations adjusting for age and BMI and fitted a multivariable linear regression of breath-holding time on WHR, age, and BMI in males. Physical activity level, fasting status, time of day of testing, and menstrual cycle phase in female participants were not recorded in the original data collection and could therefore not be included as covariates; this remains a limitation (Section 4.1).

For parameters with a statistically significant or borderline sex difference, effect size (Cohen’s d, pooled-variance method) and the approximate 95% confidence interval of the between-sex mean difference were computed from the reported means, standard deviations, and group sizes, using Student’s t distribution with n_1_ + n_2_ − 2 degrees of freedom; these values are reported to aid interpretation of the magnitude, and not merely the statistical significance, of each difference. Cohen’s d and the mean-difference confidence interval are reported throughout as male minus female, such that a positive value indicates a higher mean in males and a negative value indicates a higher mean in females (see **Table 1** footnote). Nominal and multiplicity-adjusted significance are both reported throughout, and findings that lose significance after correction, or that attenuate after adjustment for age and BMI, are interpreted as exploratory rather than confirmatory. A two-tailed p-value < 0.05 was considered nominally significant.

### 2.7 Ethical considerations

Ethical approval was obtained from the Federal University of Technology, Owerri Research Ethics Committee, Approval No. FUT/COM/SBMS/REC/Vol1/002, October 27, 2025. Written informed consent was obtained from all participants, and confidentiality was maintained throughout using anonymised participant codes.

### 2.8 Reporting guideline

This study is reported in accordance with the STROBE (STrengthening the Reporting of OBservational studies in Epidemiology) statement for cross-sectional studies (von Elm et al., 2008).

## 3 Results

Of the 81 eligible students approached for recruitment, all 81 (45 male, 36 female) agreed to participate, completed every study measurement, and were included in the final analysis; there was no non-participation, dropout, or missing data for any of the nine anthropometric and haemodynamic variables (**Table 1**).

**Table 1** summarises all nine anthropometric and haemodynamic parameters, together with *post-hoc* effect sizes and false-discovery-rate–adjusted significance (adjusted α = 0.05/9 = 0.0056 for this nine-comparison family). Waist circumference did not differ significantly between males and females, and the effect size for this difference was negligible (d = 0.19). WHR was significantly higher in males at both the nominal and the FDR-adjusted threshold (q < 0.01), with a large effect size (d = 0.86), consistent with a more android pattern of fat distribution despite equivalent absolute waist girth. Resting heart rate was significantly higher in females at both thresholds (q = 0.014; medium-to-large effect, d = −0.69, reflecting the female-higher direction). Systolic blood pressure (medium effect, d = 0.50, nominal p = 0.027, q = 0.076) and breath-holding time (small-to-medium effect, d = 0.45, nominal p = 0.047, q = 0.092) were higher in males at the nominal p < 0.05 threshold but did not remain significant after FDR correction. Waist-to-height ratio (small-to-medium effect, d = −0.43, nominal p = 0.057, q = 0.093) was higher in females and mean arterial pressure (small-to-medium effect, d = 0.44, nominal p = 0.055, q = 0.091) was higher in males, each approaching but not reaching the nominal 0.05 threshold, and neither survived correction. Waist circumference, conicity index, and diastolic blood pressure showed no meaningful sex difference at either threshold (nominal p = 0.403, 0.352, and 0.108 respectively; q = 0.403, 0.396, and 0.139). Of the nine parameters, only WHR and resting heart rate remained significant at q < 0.05. **Figures 1**–**3** illustrate the between-sex comparisons for waist circumference and WHR, systolic blood pressure and resting heart rate, and breath-holding time.

**Figure 1:**
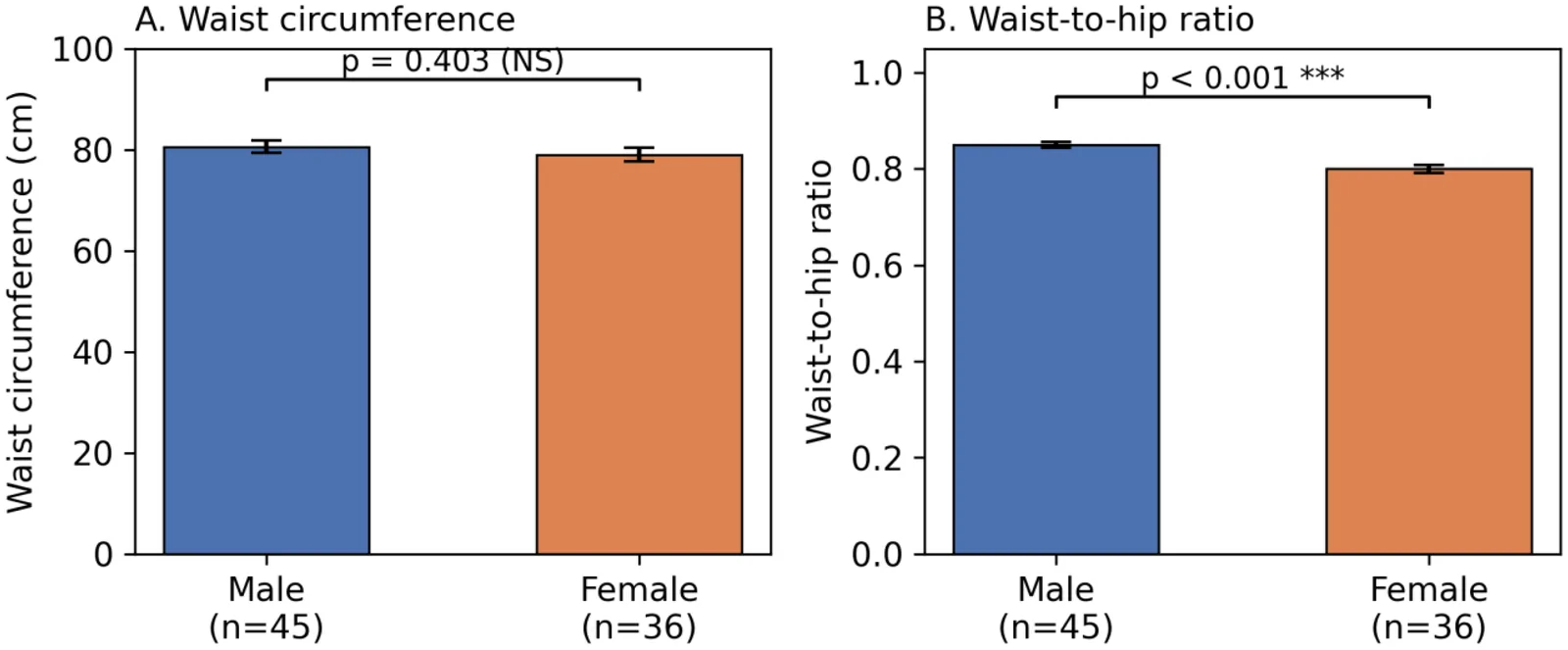
Waist circumference **(A)** and waist-to-hip ratio **(B)** by sex. Bars show mean ± SEM; SEM derived from the reported SD and n for each group. NS, not significant.

**Figure 2:**
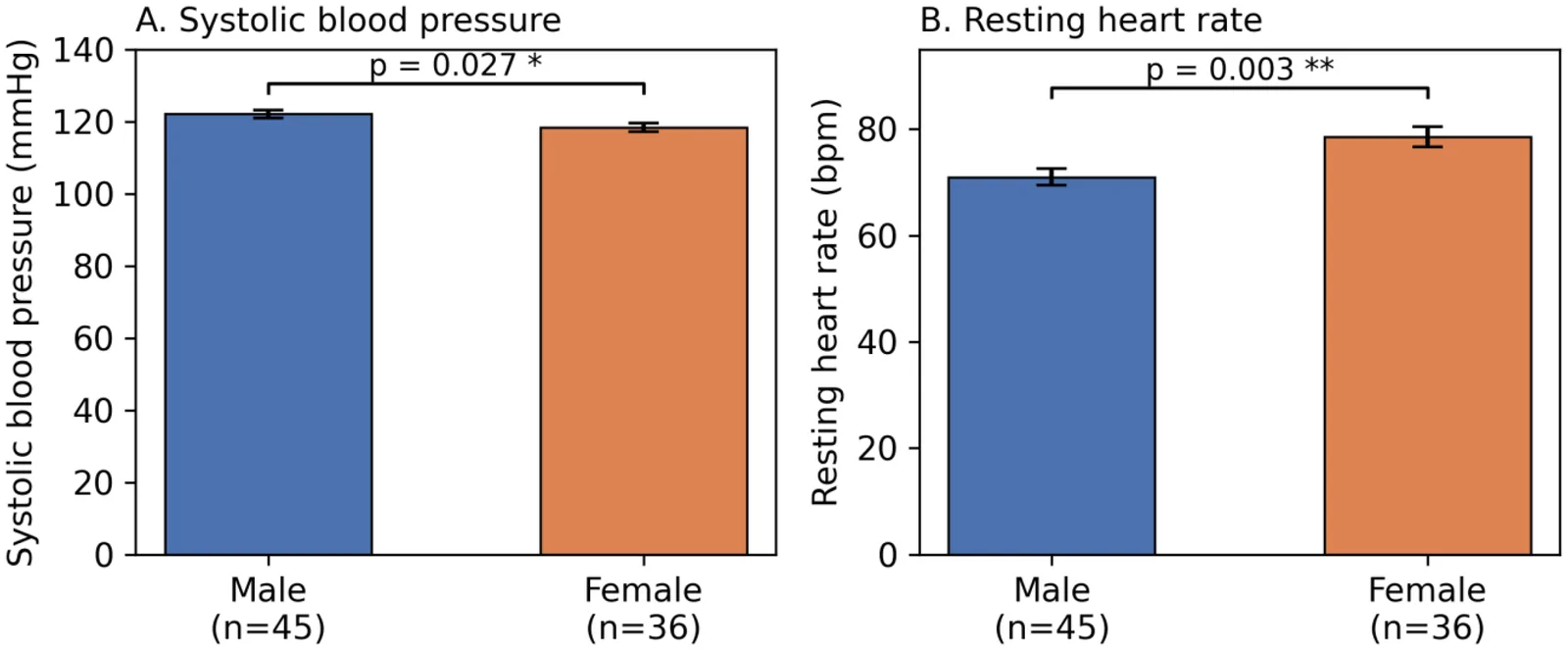
Systolic blood pressure **(A)** and resting heart rate **(B)** by sex. Bars show mean ± SEM.

**Figure 3:**
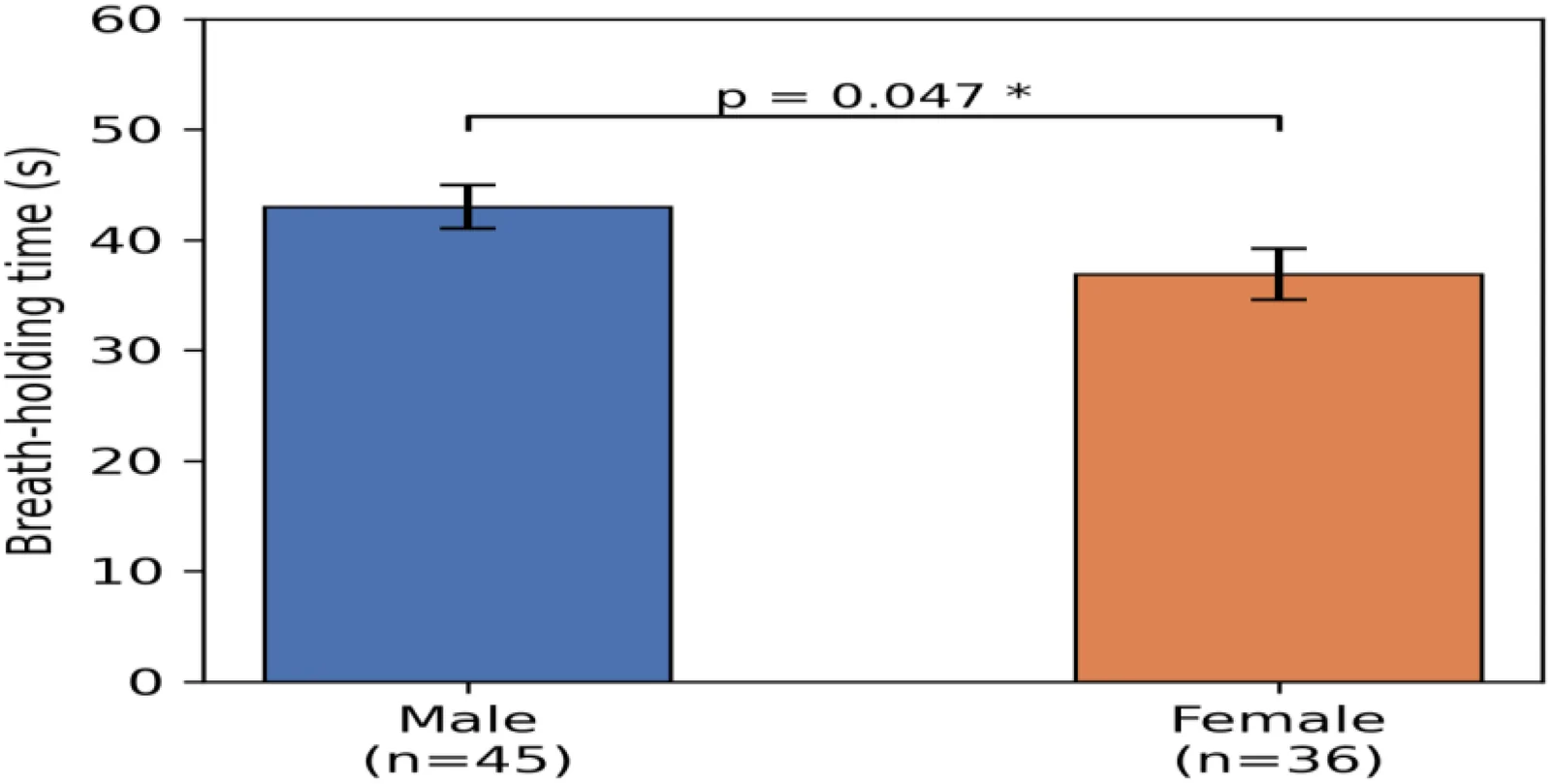
Breath-holding time by sex. Bars show mean ± SEM.

Because between-sex t-tests assume approximately normal, equally varied distributions, we formally tested these assumptions for each of the nine parameters rather than relying on the central limit theorem alone. Shapiro-Wilk testing indicated significant non-normality (p < 0.05) for waist circumference and breath-holding time in males, conicity index and both systolic and diastolic blood pressure in at least one sex, and resting heart rate in males (**Table 2**). Levene’s test indicated no significant departure from equal variances for any of the nine parameters (all p > 0.30). Mann-Whitney U tests, performed for all nine parameters as a distribution-free sensitivity analysis, agreed with the t-test-based conclusions for eight of nine variables. The exception was waist-to-height ratio, for which the Mann-Whitney U test reached nominal significance (p = 0.038) where the t-test did not (p = 0.057); this difference did not alter the overall pattern of findings, as neither test survived FDR correction for this variable (q = 0.093 and q = 0.085 respectively). We report this discrepancy transparently rather than selectively citing the more favourable test.

As an internal-consistency check, WHtR correlated very strongly with waist circumference, BMI, and hip circumference in both sexes (ρ = 0.90–0.92 with waist circumference, 0.71–0.74 with BMI, and 0.62–0.75 with hip circumference; all p < 0.001; **Table 3**, upper panel). Because WHtR is calculated directly from waist circumference, and BMI and hip circumference are close correlates of the same underlying anthropometry, this pattern is expected by mathematical construction and is not treated as an independent empirical finding or as evidence of WHtR’s discriminant validity; these three correlations are therefore excluded from the multiplicity-corrected hypothesis-testing family below.

Within the fourteen-test hypothesis-testing family (**Table 3**, lower panel), the relationship between WHR and WHtR differed by sex, reaching significance in females (ρ = 0.670, p < 0.001, surviving FDR correction) but only approaching significance in males (ρ = 0.285, p = 0.057, q = 0.201). Heart rate correlated positively with systolic blood pressure in males (ρ = 0.325, nominal p = 0.030) but not females (ρ = 0.111, p = 0.519); the male association did not survive FDR correction (q = 0.201) and should be treated as exploratory. Neither sex showed a significant correlation between heart rate and diastolic blood pressure (males ρ = 0.194, p = 0.201; females ρ = 0.171, p = 0.319).

Among males, WHR showed a modest positive correlation with breath-holding time (ρ = 0.294, nominal p = 0.050), which did not survive FDR correction (q = 0.201); no other adiposity index correlated with breath-holding time in males (waist circumference ρ = −0.056, p = 0.716; WHtR ρ = −0.073, p = 0.635; ConI ρ = 0.182, p = 0.233), and no adiposity index correlated with breath-holding time in females (ρ range −0.20 to −0.06, all p > 0.28). Exact coefficients, p-values, and bootstrap 95% confidence intervals for every correlation are reported in **Table 3**, resolving the incomplete reporting flagged in the previous round of review.

### 3.1 Confounder-adjusted sensitivity analyses

Because the sex-stratified WHR–breath-holding time correlation in males (ρ = 0.294, nominal p = 0.050) is the study’s most novel and physiologically counter-intuitive finding, we tested its robustness to adjustment for age and BMI. The partial Spearman correlation, adjusting for both covariates, was ρ = 0.279 (p = 0.063, n = 45) – materially unchanged in magnitude but no longer even nominally significant. A multivariable linear regression of breath-holding time on WHR, age, and BMI in males was not significant overall (R² = 0.089, F(3,41) = 1.34, p = 0.274), and WHR was not an independent predictor within this model (β = 64.99 s per unit WHR, 95% CI −25.12 to 155.10, p = 0.153). The heart rate–systolic blood pressure correlation in males likewise attenuated after adjustment for age and BMI (partial ρ = 0.254, p = 0.092). By contrast, the WHR–WHtR correlation in both sexes remained robust to the same adjustment (partial ρ = 0.542, p < 0.001 in males; partial ρ = 0.662, p < 0.001 in females). These adjusted analyses reinforce, rather than overturn, the cautious interpretation already given to the unadjusted WHR–BHT and heart rate–blood pressure associations in the Discussion: both are small, fragile, and should not be interpreted as evidence of a robust physiological pathway without confirmation in a larger, prospectively-adjusted sample. Physical activity level could not be included in this adjustment because it was not recorded in the original data collection (Section 4.1).

## 4 Discussion

The central finding of this study is that male and female participants with statistically indistinguishable waist circumference nonetheless differed markedly in WHR, systolic blood pressure, heart rate, and breath-holding time. This dissociation between an absolute measure of abdominal girth and a ratio-based measure of fat distribution reinforces the argument that waist circumference alone, like body mass index, may be insufficient to capture the cardiometabolic risk conferred specifically by centralised fat deposition (Janssen et al., 2004; Wildman et al., 2008; Ashwell and Gibson, 2016). This distinction has direct clinical weight: in a UK Biobank cohort of over 386,000 adults, individuals with normal waist circumference but elevated WHtR still carried materially higher cardiovascular risk than their counterparts with concordantly normal indices (Feng et al., 2024), and WHR has been shown to reclassify cardiovascular risk beyond BMI and waist circumference across nine distinct cardiovascular outcomes (Butt et al., 2023). Our data extend this waist-circumference/WHR dissociation to an apparently healthy, non-obese young adult population, where it might otherwise be assumed not yet to matter.

The nominally higher systolic blood pressure and its nominal positive correlation with heart rate in males would, if confirmed, be consistent with a hemodynamic-load pathway linking visceral adiposity to increased sympathetic activity, renin–angiotensin–aldosterone activation, and vascular resistance (Engeli et al., 2005; Hall et al., 2015); however, neither finding survived correction for multiple comparisons in this sample, both attenuated further after adjustment for age and BMI (Section 3.1), and both should be regarded as exploratory rather than as evidence of this pathway. If replicated in an adequately powered study, detecting such a pattern in normotensive, apparently healthy young men would suggest that physiological precursors of hypertension described in older or clinically obese populations (Grassi et al., 1995; Engeli et al., 2005) may already be present considerably earlier in the life course, but that inference is not supported by the present data alone. By contrast, the higher resting heart rate in females, uncorrelated with blood pressure, points to a distinct mode of cardiovascular regulation. This pattern is consistent with a broader literature on sex differences in cardiac autonomic control: women typically show higher resting heart rate alongside relatively preserved or even augmented vagally-mediated heart rate variability, while baroreflex sensitivity tends to be lower in women than in men (Huikuri et al., 1996; Koenig and Thayer, 2016), and gonadal hormones are thought to modulate sympathetic and parasympathetic balance differently by sex (Convertino, 1998). This heart-rate finding did survive correction for multiplicity and is the more robust of the two sex differences discussed here; we frame it as reflecting sex-typical autonomic regulation rather than a uniformly more or less favourable cardiovascular profile in either sex.

The respiratory findings add a further, less expected dimension, and we have substantially revised our interpretation of them in response to reviewer critique. The original framing of breath-holding time as a proxy for mechanical chest-wall restriction is not well supported: physiological work on voluntary apnoea consistently shows that breath-holding duration in healthy adults is governed primarily by an interplay of peripheral and central chemoreceptor sensitivity to carbon dioxide, prior oxygenation, and volitional tolerance, rather than by chest-wall mechanics per se (American Thoracic Society, 1995; Bosco et al., 2004; Skow et al., 2015). We therefore reframe breath-holding time here as an exploratory, integrated measure of autonomic and chemoreflex cardiopulmonary tolerance, not as a surrogate for diaphragmatic or chest-wall restriction. Read this way, our finding that WHR correlated positively, not negatively, with breath-holding time in males is genuinely counter-intuitive against the one directly comparable study we are aware of: in men with obesity, breath-holding duration was shorter, and higher WHR (alongside peripheral chemosensitivity to carbon dioxide) independently predicted the reduction in a multifactor regression model (Trembach and Zabolotskikh, 2018). A plausible reconciliation is that the relationship between central adiposity and breath-holding tolerance may not be linear across the full adiposity range: in a lean cohort such as ours (mean WHR 0.85 in males, well below thresholds conventionally used to define android obesity), WHR may covary with thoracic or muscular frame, physical conditioning, or carbon-dioxide tolerance in a way that only reverses sign once a mechanical-restriction threshold is crossed at higher adiposity. This reconciliation is speculative, however, and the finding itself did not survive correction for multiple comparisons and weakened further, to non-significance, after adjustment for age and BMI (Section 3.1); we treat it as hypothesis-generating only, and one that would need testing directly against objective chemoreflex measures (e.g., the single-breath carbon dioxide test (Trembach and Zabolotskikh, 2018)) across a wider adiposity range before any claim of a sex-specific adiposity–respiratory relationship, mechanistic or otherwise, could be entertained. The absence of any adiposity–BHT association in females may reflect a narrower range of adiposity values in this subgroup, hormonal or anatomical influences on respiratory endurance, or both.

For a respiratory audience, the practical reading of this null-to-fragile pattern is arguably as informative as a robust positive finding would have been: in a lean, apparently healthy population with a normal range of central adiposity, a simple field-deployable respiratory measure did not detect a correction-surviving or confounder-robust adiposity effect, suggesting that any mechanical or chemoreceptor-mediated impact of central fat distribution on breath-holding capacity, if present at all in this population, is small relative to inter-individual variability at this age and adiposity range. This does not exclude an effect detectable with objective spirometry, plethysmography, or a standardised single-breath carbon dioxide chemoreflex test (Trembach and Zabolotskikh, 2018), nor does it exclude an effect that emerges at higher adiposity or older age; it does suggest that breath-holding time alone, measured after a normal inspiration as in the present protocol, is unlikely to be a sufficiently sensitive early-warning tool for adiposity-related respiratory change in lean young adults, and that any future use of BHT for opportunistic respiratory screening in resource-limited settings would need validation against chemoreflex-sensitive or spirometric outcomes, using a standardised lung-volume starting point (American Thoracic Society, 1995), in a cohort spanning a wider adiposity range before that practical application could be recommended.

WHtR correlated very strongly with waist circumference, body mass index, and hip circumference in both sexes. We are deliberately brief in interpreting this: because WHtR is calculated directly from waist circumference, and shares further determinants with BMI and hip circumference, this correlation is expected by mathematical construction and does not, by itself, demonstrate that WHtR outperforms body mass index or waist circumference as a screening index (Janssen et al., 2004; Ashwell et al., 2012; Ashwell and Gibson, 2016). Evaluating that comparative question properly requires testing WHtR’s association with an outcome that is independent of its own input variables – an imaging-based visceral fat measure, or a hard cardiometabolic endpoint – rather than with other anthropometric ratios that share its own inputs; large external cohorts that have done so generally support WHtR and WHR as incrementally useful over BMI (Ashwell et al., 2012; Butt et al., 2023; Feng et al., 2024), but our internal-consistency correlations cannot themselves adjudicate this. We therefore centre our own comparative discussion on the dissociation between absolute waist circumference and WHR (which do not share the same information content, as shown by their divergent between-sex patterns in **Table 1**) rather than on WHtR’s correlation with its own component variables. WHtR showed a between-sex difference (higher in females) that approached but did not reach nominal significance (p = 0.057 by t-test; p = 0.038 by Mann-Whitney U) and did not survive FDR correction by either test; this cohort therefore provides no evidence, in either direction, that WHtR outperforms simpler indices for screening purposes in this population.

### 4.1 Limitations

This study has several limitations. Its cross-sectional design precludes causal inference between adiposity and hemodynamic or respiratory outcomes. The sample was drawn from a single university and restricted to an apparently healthy 18–30-year age range, limiting generalisability; participants were selected by random/systematic sampling of the eligible population, but no *a priori* sample size or power calculation is reported. With N = 81 (45 men, 36 women), the study is underpowered to detect small effects, and the sex differences and correlations that were significant only at the nominal, uncorrected threshold (systolic blood pressure, breath-holding time, waist-to-height ratio by Mann-Whitney U, heart rate–blood pressure correlation, WHR–breath-holding time correlation) should be treated as preliminary until replicated in an adequately powered sample. Because WHR, WHtR, and conicity index all share waist circumference as an input variable, correlations among these indices are partly definitional and cannot be interpreted as independent validation of one another; we have accordingly reported the WHtR–waist circumference/BMI/hip circumference correlations separately from the corrected hypothesis-testing family (Section 2.6, **Table 3**).

Between-sex comparisons of continuous variables were performed using independent-samples t-tests; for this revision we formally tested the underlying assumptions rather than relying on sample size alone. Shapiro-Wilk testing indicated significant non-normality for several variables (notably waist circumference and breath-holding time in males, and blood pressure variables in one or both sexes; **Table 2**), and Mann-Whitney U tests were therefore performed as a distribution-free sensitivity analysis for all nine between-sex comparisons; conclusions were consistent with the t-test-based results for eight of nine variables, with waist-to-height ratio the sole exception (Section 3, **Table 2**). Levene’s testing indicated no significant departure from homogeneity of variance for any variable. Exact rank-correlation coefficients, p-values, and bootstrap 95% confidence intervals are now reported for every correlation in **Table 3**, resolving the incomplete reporting in the previous draft; partial correlations and a multivariable regression additionally adjusting for age and BMI are reported in Section 3.1 for the two correlations of greatest interest. Physical activity level, fasting status, time of day of testing, and menstrual cycle phase in female participants were not recorded in the original data collection and therefore could not be included as covariates in these adjusted analyses; all reported associations should be understood as unadjusted for these factors and potentially confounded by them.

Breath-holding time is a simple volitional measure influenced by motivation and familiarity with the procedure and was not corroborated with objective spirometric, plethysmographic, or chemoreflex indices of respiratory function (e.g., the single-breath carbon dioxide test (Trembach and Zabolotskikh, 2018)); although standardized verbal instructions and an explicit safety-stop protocol were used (Section 2.5), readers should interpret the respiratory findings as hypothesis-generating rather than definitive evidence of altered pulmonary mechanics or chemoreflex sensitivity. A further, specific limitation – raised directly by peer review – is that breath-holding was initiated after a normal (tidal) inspiration rather than at a standardised lung volume such as total lung capacity or functional residual capacity; because breath-holding duration is itself sensitive to starting lung volume (American Thoracic Society, 1995; Bosco et al., 2004; Skow et al., 2015), this introduces intra- and inter-individual variance in our BHT measurements that is unrelated to adiposity or chemoreceptor sensitivity, and that a standardised-lung-volume protocol would eliminate in future replications. Blood pressure was measured manually rather than with ambulatory or automated devices, and visceral fat was estimated indirectly from anthropometry rather than imaging. Biochemical markers of inflammation and insulin resistance were not assessed, and no statistical adjustment for age within the 18–30 range was possible beyond the partial correlation and regression analyses reported in Section 3.1.

## 5 Conclusion

In this cohort of healthy young Nigerian adults, WHR was robustly higher in males than females independent of absolute waist circumference, and resting heart rate was robustly higher in females – both differences surviving correction for multiple comparisons. Sex differences in systolic blood pressure and breath-holding time, and a correlation between WHR and breath-holding time in males, were observed at nominal significance but not after correction for multiple comparisons, and the WHR–breath-holding time association weakened further to non-significance after adjustment for age and BMI; these findings should be treated as preliminary. We have reframed breath-holding time as a measure of integrated autonomic and chemoreflex cardiopulmonary tolerance rather than a proxy for mechanical chest-wall restriction, consistent with the physiological literature on voluntary apnoea. These findings support further investigation of simple, low-cost adiposity indices – particularly WHR – for early cardiometabolic screening of young adults, and highlight the need for adequately powered, multi-centre longitudinal studies incorporating objective pulmonary and chemoreflex function testing, imaging-based fat quantification, and standardised covariate capture (physical activity, fasting status, menstrual cycle phase) before any adiposity–respiratory relationship, sex-specific or otherwise, can be considered established.

## Data Availability

The raw data supporting the conclusions of this article will be made available by the authors, without undue reservation.
